# Known unknowns in an imperfect world: incorporating uncertainty in recruitment estimates using multi-event capture–recapture models

**DOI:** 10.1002/ece3.846

**Published:** 2013-10-25

**Authors:** Marine Desprez, Clive R McMahon, Mark A Hindell, Robert Harcourt, Olivier Gimenez

**Affiliations:** 1 Marine Predator Research Group, Department of Biological Sciences, Macquarie UniversityNorth Ryde, 2109, New South Wales, Australia; 2 Institute for Marine and Antarctic Studies, University of TasmaniaHobart, 7001, Tasmania, Australia; 3 Centre d'Ecologie Fonctionnelle et Evolutive, campus CNRS, UMR 51751919 Route de Mende, Montpellier Cedex 5, 34293, France

**Keywords:** breeding state assignment, multistate capture–recapture models, primiparity, southern elephant seals, state uncertainty, vital rates

## Abstract

Studying the demography of wild animals remains challenging as several of the critical parts of their life history may be difficult to observe in the field. In particular, determining with certainty when an individual breeds for the first time is not always obvious. This can be problematic because uncertainty about the transition from a prebreeder to a breeder state – recruitment – leads to uncertainty in vital rate estimates and in turn in population projection models. To avoid this issue, the common practice is to discard imperfect data from the analyses. However, this practice can generate a bias in vital rate estimates if uncertainty is related to a specific component of the population and reduces the sample size of the dataset and consequently the statistical power to detect effects of biological interest. Here, we compared the demographic parameters assessed from a standard multistate capture–recapture approach to the estimates obtained from the newly developed multi-event framework that specifically accounts for uncertainty in state assessment. Using a comprehensive longitudinal dataset on southern elephant seals, we demonstrated that the multi-event model enabled us to use all the data collected (6639 capture–recapture histories vs. 4179 with the multistate model) by accounting for uncertainty in breeding states, thereby increasing the precision and accuracy of the demographic parameter estimates. The multi-event model allowed us to incorporate imperfect data into demographic analyses. The gain in precision obtained has important implications in the conservation and management of species because limiting uncertainty around vital rates will permit predicting population viability with greater accuracy.

## Introduction

Estimating demographic parameters is fundamental to understand animal population dynamics and investigating life-history strategies (Caswell 2001; Morris and Doak 2002; Williams et al. 2002). Incorrect estimates of demographic parameters, in particular age of first reproduction, can lead to biased estimates of fitness, flawed inferences about population viability (Patterson and Murray 2008) and make the detection of evolutionary trade-offs difficult (Cam et al. 2002; Buoro et al. 2012). However, identifying an individual's reproductive status in the field is not always possible. In particular, determining when an individual breeds for the first time can be difficult when the probability of detection within a year is less than one (Buoro et al. 2010). In many birds and mammals, young and inexperienced individuals breeding for the first time have less chance of being successful compared with more experienced breeders or individuals that delay their first reproductive event to an older age (Cam and Monnat 2000; Hadley et al. 2006, 2007; Sanz-Aguilar et al. 2008, 2009; Limmer and Becker 2010). Consequently, young first-time breeders are likely to abort, abandon their offspring or give birth to offspring that do not survive long enough to be detected. Under these circumstances, individuals may be wrongly considered nonbreeders leading to a biased estimate of the age at first reproduction. To avoid making this error, the conservative approach is to analyze only data from individuals whose reproductive status has been determined with certainty, and this has been the established practice. However, doing so reduces the sample size of the dataset, thereby decreasing the statistical power to detect signals of biological importance and potentially introduces bias in the estimates of age of first reproduction.

Multistate capture–recapture models (MSM) are widely used to estimate demographic parameters such as survival (Lebreton et al. 2009) and transition probabilities between breeding states (Nichols et al. 1994; Cam et al. 1998; Barbraud and Weimerskirch 2005; Crespin et al. 2006; Sanz-Aguilar et al. 2008) while accounting for the fact that the probability of detecting an individual in the wild is less than one. Ignoring imperfect detection can lead to biased estimates and flawed inference (Gimenez et al. 2008) but this is often not the only source of uncertainty in capture–recapture studies (Pradel 2009). Even when an individual is observed in the field, its status can still remain unknown or uncertain [*e.g.,* sex (Nichols et al. 2004; Pradel et al. 2008; Genovart et al. 2012), epidemiologic status (Conn and Cooch 2009), reproductive status (Gimenez et al. 2012)]. To deal with this issue and to allow the use of imperfect field data, an extension of the multistate capture–recapture framework, known as the multi-event model (MEM) (Pradel 2005), has been developed. Besides accounting for imperfect detection, this model also accounts for uncertainties in the assessment of state. The MEM therefore allows the use of all the data collected unlike the MSM that forces a reduction in the sample and potentially removes a whole segment of the population. The MEM framework has already been used to assess, among other things, the probability of skipping reproduction (Sanz-Aguilar et al. 2011), the influence of reproductive experience on breeding probabilities (Desprez et al. 2011) and to estimate demographic parameters while accounting for mark loss (Juillet et al. 2011) (see Gimenez et al. 2012 for a detailed review). To date, however, no studies aiming to estimate recruitment probabilities while specifically accounting for uncertainty in breeding status have been undertaken.

Estimating demographic parameters from both MSM and MEM requires adequate capture–recapture data and annual observations of reproductive status. In this regard, the Macquarie island population of southern elephant seal (*Mirounga leonina*) provides an ideal study population as a large number of known-age animals have been uniquely marked and resighted. However, the first breeding event in an elephant seal's life remains difficult to observe and record with certainty. This is in part because the end of the breeding season overlaps with the beginning of the juvenile molting period. Accordingly, it is not always possible to distinguish between a young seal coming ashore for its first breeding event from a seal hauling out for its annual molt. Moreover, young sexually mature males, even if still too small and inexperienced to compete in harems (*i.e.,* they are socially immature), often remain on the beaches trying to mate. Copulations involving these males are rarely observed but may still be successful and produce offspring (Fabiani et al. 2004). For the females, the presence of a pup in close proximity is often taken as a proof of their breeding status but if first-time breeders lose their pup prepartum or early postpartum, they may wrongly be considered nonbreeders due to the absence of a pup. Consequently, making the distinction between a juvenile (an individual that has not bred yet) and a first-time breeder is not always obvious.

Here, we used a MEM framework to assess survival and recruitment, from data including individuals for which the breeding state was unknown on one or several occasions. We compared these estimates to those obtained from a standard MSM capture–recapture analysis, in which data from individuals with known breeding status only (juveniles or adults) were analyzed. In particular, we quantified the gain in precision obtained from the use of data including uncertainties by comparing the standard errors of the same parameter estimates obtained under MSM and MEM.

## Materials and Methods

### Introduction to the study species

Southern elephant seals (*Mirounga leonina*) have a circumpolar distribution in the Southern Ocean (McMahon et al. 2005). While they spend most of their lives at sea foraging, they return to land biannually, once to molt (timing depending on sex and age (Hindell and Burton 1988)) and once to breed (September–November).

Each year from 1993 to 1999, approximately 2000 recently weaned southern elephant seals were permanently and uniquely marked with hot iron brands (McMahon et al. 2006b) at Macquarie Island (54°30′S, 158°50′E). Although elephant seals travel long distances to forage, the Macquarie Island population is considered a closed breeding population and is the only major Pacific sector breeding population in the Southern Ocean (McMahon et al. 2005). Until 2001, intensive searches were made for branded individuals (daily searches on the isthmus, the main study area, and the area to which most seals return (McMahon et al. 2003); every 10 days around the top third of the island and once a month around the whole island). Despite this intensive effort, the first breeding event in an elephant seal's life remained difficult to observe and record with certainty. From 2001 onwards, resightings were opportunistic according to availability of personnel.

Our aim was to model the most uncertain part (first breeding events) of the life cycle of elephant seals. As a large proportion of the males die before reaching this step, we analyzed only the data from female seals. To coincide with the southern elephant seal life cycle, we considered that a year started in September and ended in August (*e.g.,* the first year of our study runs from September 1993 to August 1994, hereafter referred to as 1993). We considered two breeding states: the juvenile state (individuals that have not bred yet) and the adult state (seals that have bred at least once). We determined the breeding state of a female according to (1) the age [all females from 0 to 2 years old were considered juveniles because recruitment never occurred before 3 years of age (McMahon et al. 2003)], (2) the presence of a pup with the female (any individual seen with a pup was considered an adult), and (3) the period during which the female was seen ashore (details in Appendix S1). All females considered “adults” on one occasion were then considered “adults” for the rest of their life. An “unknown” status was assigned each time a breeding state could not be assigned using one of the above criteria.

### Multistate capture–recapture model (MSM)

The standard capture–recapture model used to estimate recruitment probabilities while accounting for imperfect detection was a multistate model (Lebreton et al. 2009) with three states: juveniles (J), adults (A), and dead individuals (D) underlying three observations or events: (1) not seen; (2) seen as juvenile; (3) seen as adult. The breeding status was known with certainty for each individual and each sampling occasion. This model included three parameters: resighting probability (*p*) that linked the observations made in the field to the breeding states, survival probability (Φ), and transition probability between states (*i.e.,* recruitment) (ψ). The observation process and the temporal dynamic of states could be summarized in the matrix of resighting probabilities *P*, with states at *t* in rows and observations at *t* in columns, and matrices of survival *S* and transition *T*, with states at *t* in rows and states at *t + 1* in columns:


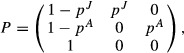



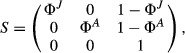



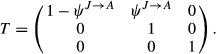


For instance, a juvenile had a probability *p*^J^ of being resighted at time *t* (matrix *P*, 1st row, 2nd column) and the complementary probability (1 − *p*^J^) not to be seen (matrix *P*, 1st row, 1st column),whereas its probability of being resighted as an adult was null and fixed to 0 (matrix *P*, 1st row, 3rd column). Then, this individual could either survive from time *t* to *t + 1* with a probability Ф^J^ (matrix *S*, 1st row, 1st column) or die with a probability 1 − Ф^J^ (matrix *S*, 1st row, last column). Finally, it could either breed with a probability of ψ^*J*→*A*^ and become an adult at *t + 1* (matrix *T*, 1st row, 2nd column) or remain juvenile with a probability 1 − ψ^*J*→*A*^ (matrix *T*, 1st row, 1st column). A dead individual, however, could not be seen. Its probability of being resighted was thus fixed to 0 (last row, 2nd and 3rd columns). Its survival probability from time *t* to *t + 1* was also null and fixed to 0 (matrix *S*, last row, 1st and 2nd columns) as well as its transition probability to another state (matrix *T*, last row, 1st and 2nd columns).

### Multi-event capture–recapture model (MEM)

To account for uncertainties in the breeding status, we used a multi-event model (Pradel 2005) in which we considered all the possible observations made in the field during a breeding season: an individual may be missed (not seen); seen and assigned as a juvenile; seen with an unknown state; and seen and assigned as an adult. States remained the same as in the previous model, that is, juvenile, adult, and dead but, in contrast to the MSM in which there was a strict correspondence between observations and states, several observations might correspond to a single state in the MEM. In the observation process, in addition to the resighting probability, we included the probability of state assignment (β) defined as the probability that a reproductive status was assigned with certainty to an individual. The observation process was thus represented by the product of the resighting matrix (*P*) and the breeding state ascertainment matrix (*A*). Columns of the resighting matrix and rows of the breeding state ascertainment matrix corresponded to the events “individual not seen,” “juvenile detected,” and “adult detected,” whereas columns of the breeding state ascertainment matrix corresponded to the four possible observations made in the field (individual not seen; seen and assigned as a juvenile; seen with an unknown state; and seen and assigned as an adult):


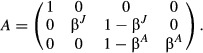


When an individual was seen during a sampling occasion with an unknown breeding status, the model considered all the possible histories. For instance, let us assume that we have 4 sampling occasions, for simplicity, and all individuals are marked as juveniles. We consider an individual with the encounter history 1123 that was marked as a juvenile (1), resighted in the second occasion as juvenile (1), seen in the third occasion in an unknown state (2), and finally seen as adult (3). Because the breeding status of this individual was unknown during the second occasion, the time of its recruitment is uncertain. Two scenarios are possible: (1) this individual was a juvenile when it was observed with an unknown status, and the probability is Φ^*J*^(1 − ψ^*J*→*A*^) *p*^J^β^J^Φ^*J*^(1 − ψ^*J*→*A*^) *p*^J^(1-β^J^) Φ^*J*^ ψ^*J*→*A*^) *p*^A^β^A^, (2) it was an adult, and the probability is Φ^*J*^(1 − ψ^*J*→*A*^) *p*^J^β^J^Φ^*J*^(1 − ψ^*J*→*A*^) *p*^A^(1-β^A^) Φ^A^*p*^A^β^A^. These two events being mutually exclusive, the probability for this particular history is the sum of the two possible probabilities.

### GOF test

Goodness of fit (GOF) tests are not available for capture–recapture models with permanent transitions (from juveniles to adults for both models and from juveniles to unknown state and unknown state to adults for the MEM) (Pradel et al. 2005). We assumed that if there was some lack of fit in the MSM, it would affect the MEM in the same way and would not compromise the comparison.

### Model selection

For the MSM, we used data consisting of capture–recapture histories from 4179 individuals for which the breeding state was always known with certainty. In the MEM, we analyzed all the 6639 capture–recapture histories including 2460 histories with one or more occasions for which an observed seal's breeding status was unknown.

For both MSM and MEM, we fitted a set of models incorporating relevant combinations of temporal and individual effects on each parameter (*p,* Φ, ψ, and β when applicable) sequentially while constraints on remaining parameters were held constant. As the sampling design varied over the study period, we considered an effect of time (representing the temporal variation between sampling periods, *i.e.,* 1 year) on the resighting and state assignment probabilities. We also investigated a state effect on the resighting probability given that juveniles avoid hauling out during the breeding season (Hindell and Burton 1988) and were thus less likely to be detected than adults. Assigning a breeding state to female elephant seals was particularly challenging for individuals between 3 and 5 years old. We thus considered, in addition to the temporal variation, an age and state effect on the state assignment probability. We also examined the state and age effects on the survival probability as we expected lower survival for young juveniles than for older individuals (McMahon et al. 2003). Regarding temporal effects on the survival and recruitment probabilities, we considered a year effect. As adult survival in long-lived iteroparous species is more likely to remain stable overtime than juvenile survival (Gaillard and Yoccoz 2003), we also examined the case in which only juvenile survival was affected by the time. Finally, we investigated the variability of recruitment probability according to the age of females. Once the main effect was determined for a parameter, we added each of the remaining effects in an additive and interactive fashion to assess whether one of these combinations was relevant. We repeated this until no better model was selected. For the MSM, we started by identifying the most appropriate structure for *p*, then for Φ, and finally for ψ using the structure for *p* and Φ selected in the previous step. For MEM, we proceeded in the same way starting by identifying the structure for β, then for *p* and Φ, and finally for ψ. We selected the most parsimonious model using the Akaike Information Criterion (AIC) (Burnham and Anderson 2002). Analyses were performed using E-SURGE (Choquet et al. 2009).

## Results

The best combination of effects influencing survival, recruitment, and resighting probabilities was the same in both MSM and MEM (Table 1). Using the method of Choquet and Cole (2012), we noticed that the recruitment parameter (varying with age and time) was not identifiable in the most parsimonious model for both MSM and MEM. Consequently, we considered the model in which recruitment depended only upon age but was identifiable. We checked that survival and resighting probabilities obtained from this model were comparable to the ones estimated from the initial best model.

**Table 1:** Model selection results for (a) the standard multistate capture–recapture model and (b) the multi-event capture–recapture model

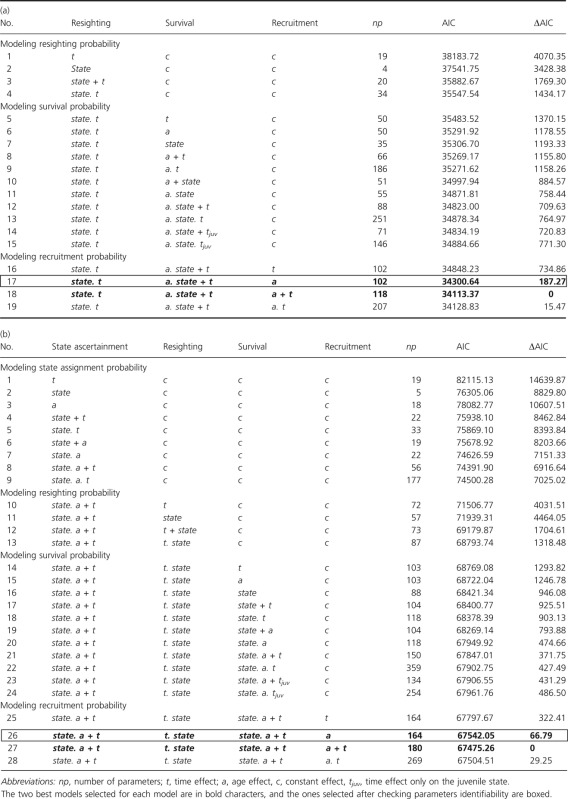

**Figure 1:**
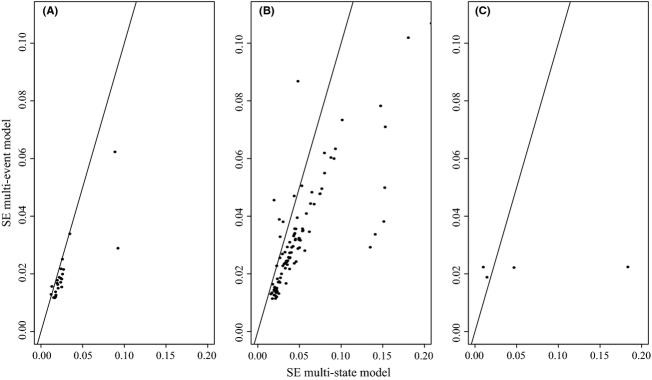
Standard errors for (A) resighting probabilities, (B) survival probabilities, and (C) recruitment probabilities of female elephants seals: from the Multi State Model vs. the Multi Event Model. Data points correspond to parameter estimates. The solid line represents the situation in which the SEs are equal for both models parameter estimates.

All parameters (except recruitment) were influenced by temporal variation. In addition to this time effect, resighting and survival probabilities varied according to the breeding state of the seals. Both survival and recruitment probabilities also depended on the age of individuals. Importantly, the MEM allowed a gain in precision for the estimates of resighting and survival as the standard errors for these parameters were lower in the MEM than in the MSM (Fig. 1). For recruitment, the standard errors obtained for the older ages (5 and 6 year old) were also lower from the MEM but not for the younger ages (3 and 4 year old) (Fig. 1).

**Figure 2:**
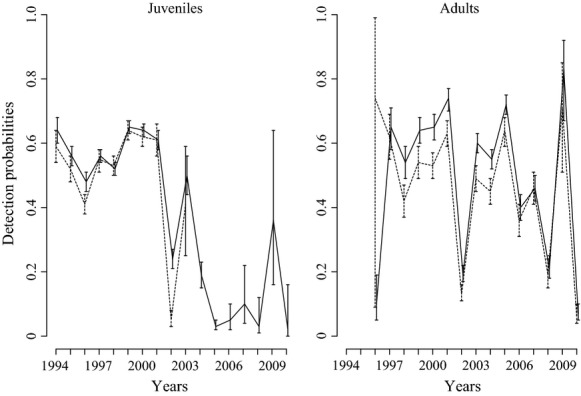
Resighting probabilities of female elephant seals by state, time, and type of capture–recapture model. The dotted line represents estimates from the MSM, and the solid line represents estimates from the MEM. First resighting event occurred in 1994 for juveniles and 1996 for adults. Estimates on the boundary are not represented.

Resighting probabilities varied with both breeding state and time, with marked fluctuations over the study period. Estimates from the MEM were higher than the ones obtained from the MSM (except for the resighting probabilities of juveniles in 1998 and for adults in 1996) (Fig. 2). From the MSM, resighting probabilities of juveniles were estimated on the boundary from 2004 whereas they were assessed until 2010 from the MEM. For adult resighting probabilities, the trend over years was the same for both MSM and MEM (except in 1996) with very low probabilities in 2002, 2008, and 2010 (Fig. 2).

**Figure 3:**
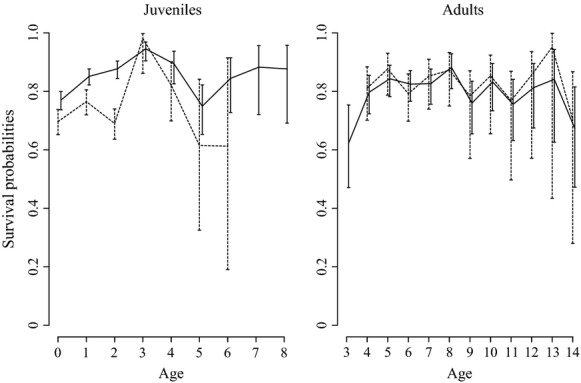
Survival probabilities of female elephant seals by state, age, and type of capture–recapture model. Each point shows the survival probability of a specific age averaged over the years. Estimates on the boundary are not represented. The dotted line represents estimates from the MSM, and the solid line represents estimates from the MEM.

**Figure 4:**
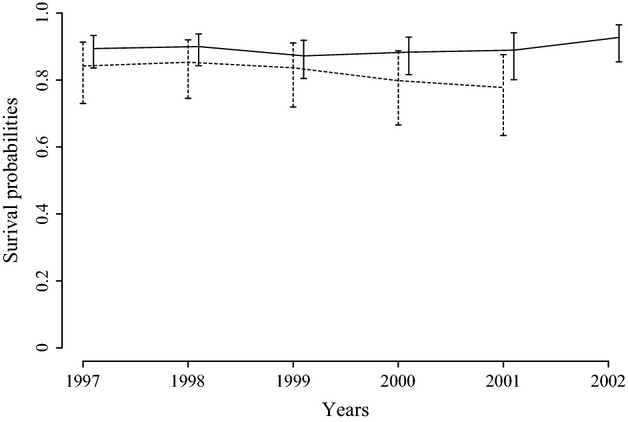
Survival probabilities of the 4-year-old female juveniles by year and type of capture–recapture model. Estimates on the boundary are not represented. The dotted line represents estimates from the MSM, and the solid line represents estimates from the MEM.

Survival probabilities depended on breeding state, age, and time (Table 1). For the juveniles, probabilities were lower when estimated from the MSM (except for the 3-year-old individuals) (Fig. 3). The use of the MEM enabled us to estimate the survival of juveniles until 2002 and for seals up to 8 years old, whereas probabilities could not be estimated after 2001 or for seals older than 6 with the MSM (Figs 3 and 4). However, the confidence intervals for the survival probabilities obtained from the MEM for seals of 7 and 8 years old were large. Concerning adult survival, the difference between the two models was smaller than for juveniles (Fig. 3) apart from the survival probability of the 3-year-olds that could not be estimated in the MSM. For both models, no survival probabilities could be estimated in 2009 or for seals older than 14 years old.

**Figure 5:**
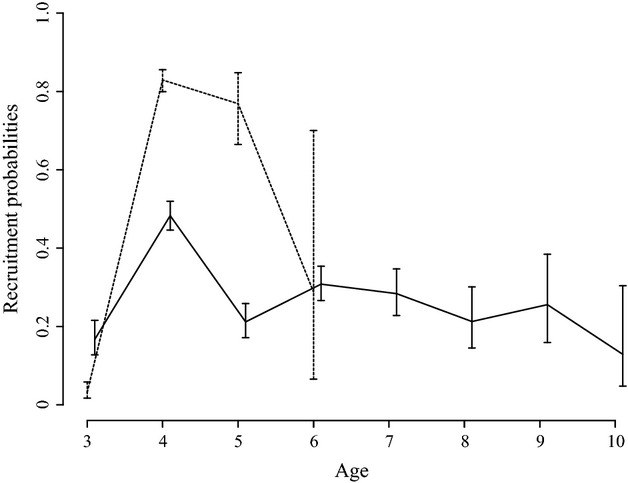
Recruitment probabilities of female elephant seals by age and type of capture–recapture model. Each point shows the probability of recruiting at a particular age averaged over the years. Estimates on the boundary are not represented. The dotted line represents estimates from the MSM, and the solid line represents estimates from the MEM.

Recruitment was influenced by age (Table 1). For both models, female elephant seals had the highest probability of recruiting at age 4 (Fig. 5). As for survival, the MEM made it possible to estimate recruitment probabilities for older individuals (10 year old *vs*. 6 year old, Fig. 5) than the MSM. Probabilities from the MEM were lower than the ones obtained from the MSM (except for the 3-year-olds) with a pronounced difference for the recruitment estimates of the 4- and 5-year-old seals (Fig. 5). Recruitment at 3 years old was low for both models.

State assignment probabilities were only estimated in the MEM and depended upon state, age, and time. However, probabilities to assign the juvenile state were not identifiable. All adults detected and older than 5 years were recorded as “adults” with certainty. Uncertainty about the adult state was very high for 3-year-old individuals.

## Discussion

Estimating precise demographic parameters, such as recruitment and survival, is of fundamental importance to the study of population dynamics and is needed to provide robust population projections (Lebreton et al. 1992; Caswell 2001). Here, by comparing estimates obtained from two different capture–recapture models, the recently developed multi-event model that explicitly accounts for uncertainty in the breeding state of the individuals, and the more standard multistate model commonly used by ecologists, we show that exploiting data including uncertainty in breeding status can greatly improve the precision and accuracy of the estimates.

Accounting for uncertainty did not affect the structure of the most parsimonious model because the demographic parameters obtained from the MEM and the MSM were influenced by the same combination of effects. However, the precision of the survival and resighting probabilities was higher in the MEM. Indeed, as it has already been reported earlier (Pradel et al. 2008; Genovart et al. 2012), considering capture–recapture histories including both certain and uncertain states can raise the size of the sampled population leading to more precise and accurate estimates. The gain in precision was less obvious when estimating recruitment probability. This is probably due to the fact that uncertainties were directly related to the recruitment parameter and concentrated on the 3- and 4-year-olds. Thus, addition of unknown breeding states in the data slightly reduced the precision of the recruitment estimates for these ages. The difference in recruitment estimates between the two models was more pronounced for the 4- and 5-year-olds with probability estimates much lower in the MEM than in the MSM. This is consistent with the fact that only two breeding states (juveniles and adults) were considered in the MSM and the number of seals assigned “juveniles” with certainty was low (<200 at 4 years old and <20 at 5 years old), while the number of adults recorded at these ages was comparatively high (≈ 1000 seals) leading to high probabilities of recruitment. In the MEM, the number of seals assigned to a breeding state was counterbalanced by the number of “unknown” seals (≈ 680 for the 4 years old and ≈ 430 for the 5 years old) that might still be juveniles. Recruitment estimates for the 4- and 5-year-old seals were thus reduced in the MEM.

In real-world datasets, uncertain field observations often constitute the bulk of the information collected (Nakagawa and Freckleton 2008; Pradel 2009), and particular statistical tools are therefore needed to exploit these data. The MEM, by accounting for uncertainties in breeding status, enabled us to use all the information available and to assess demographic parameters for longer periods and for more age classes than in the standard approach. This may be of particular importance in studies aiming to determine the influence of environmental factors on demographic parameters over time (Nevoux et al. 2010) or to investigate senescence or other trade-offs involving age (Hadley et al. 2006; Clutton-Brock and Sheldon 2010). However, it is important to note that even though using MEM improved the precision of most estimates, no accurate results could be obtained when the data only included individuals with uncertain breeding states or when resighting probabilities were very low. Consequently, determining and then maintaining an appropriate and constant sampling effort remains of paramount importance in demographic studies (Kendall et al. 2009; Clutton-Brock and Sheldon 2010; Magurran et al. 2010). This point is clearly illustrated in our study as a lot of uncertainties were induced by changes in the sampling effort for reasons beyond our control (McMahon et al. 2006a) (from an intense, systematic resighting effort to an opportunistic one, cessation of permanent marking in 1999, and severe restrictions imposed on resighting effort in 2002).

Despite this limitation, the present modeling greatly increased the precision of most of the demographic parameter estimates. This clearly illustrates the importance of including uncertainty in models for conservation and management of wildlife. Being able to include more precise demographic information in population projection models greatly enhances the ability to produce precise and reliable projected population growth rates (Caswell 2001). This is especially important in the case of species of conservation concern such as the southern elephant seal, for which accurate assessment of population viability is critical but not straightforward. In fact, for many endangered or vulnerable populations, life-history datasets are incomplete, sparse and sporadic and this will lead to imprecise vital rate estimates and subsequently uncertain assessment of population viability. This in turn may lead to inappropriate or even deleterious management decisions. We suggest that using the MEM to improve the precision of demographic parameter estimates will limit uncertainty in population projection models and so improve the reliability of conservation measures.

In conclusion, the MEM increased the precision and accuracy of our demographic parameter estimates showing that imperfect data can be usefully and successfully incorporated into demographic analyses and should not be discarded. However, while using the MEM greatly enhances our ability to deal with uncertainty, such analytical advances cannot replace appropriate sampling effort, and this still remains of paramount importance for studies aiming to quantify vital rates.

## Data Accessibility

Sightings database of Macquarie Island Elephant Seals: Australian Antarctic Data Centre (https://data.aad.gov.au/), entry ID: AADC-00102.

## Supporting Information

**Appendix S1.** Females state assignment according to haul-out dates.

**Figure S1.** Number of female elephant seals of 4 years of age resighted ashore at Macquarie Island over a 60-year period (1951–2011).

**Figure S2.** Breeding season and molting period for 4-year-old female elephant seals at Macquarie Island.

**Table S1.** Dates delimiting the breeding season and the molting period of adult females elephant seals according to their age.

**Table S2.** Minimum return date to land of adult females according to their age.
