# Supplementary material for: Known unknowns in an imperfect world: incorporating uncertainty in recruitment estimates using multi-event capture–recapture models
Source: Ecol Evol. 2013 Oct 25;3(14):4658–68. doi: 10.1002/ece3.846 (PMC3867902; doi:10.1002/ece3.846)
Supplement: Supplementary file 1 [file ece30003-4658-SD1.docx]

**Appendix 1. Females state assignment according to haul-out dates**

Southern elephant seals have a predictable annual haul-out lifecycle characterized by three periods ashore: the breeding season, the moulting period and the mid-year haul-out (mostly for juveniles). We represented the number of females resighted ashore from September to August (to coincide with the elephant seals lifecycle) at Macquarie Island over a 60 years period (1951 - 2011) for each age from 3 (age at which the youngest females breeders are detected) to 12 years old. No representation was done for older seals because of the very low number of females resighted. The number of seals ashore during most haul-outs closely approximated a normal distribution ([Hindell and Burton 1988](#_ENREF_1)). We used a generalized additive model GAM ([Wood 2006](#_ENREF_4)) to determine the curve that best fitted the data. We represented the upper and lower 95% confidence interval of this curve (Fig.1).

**
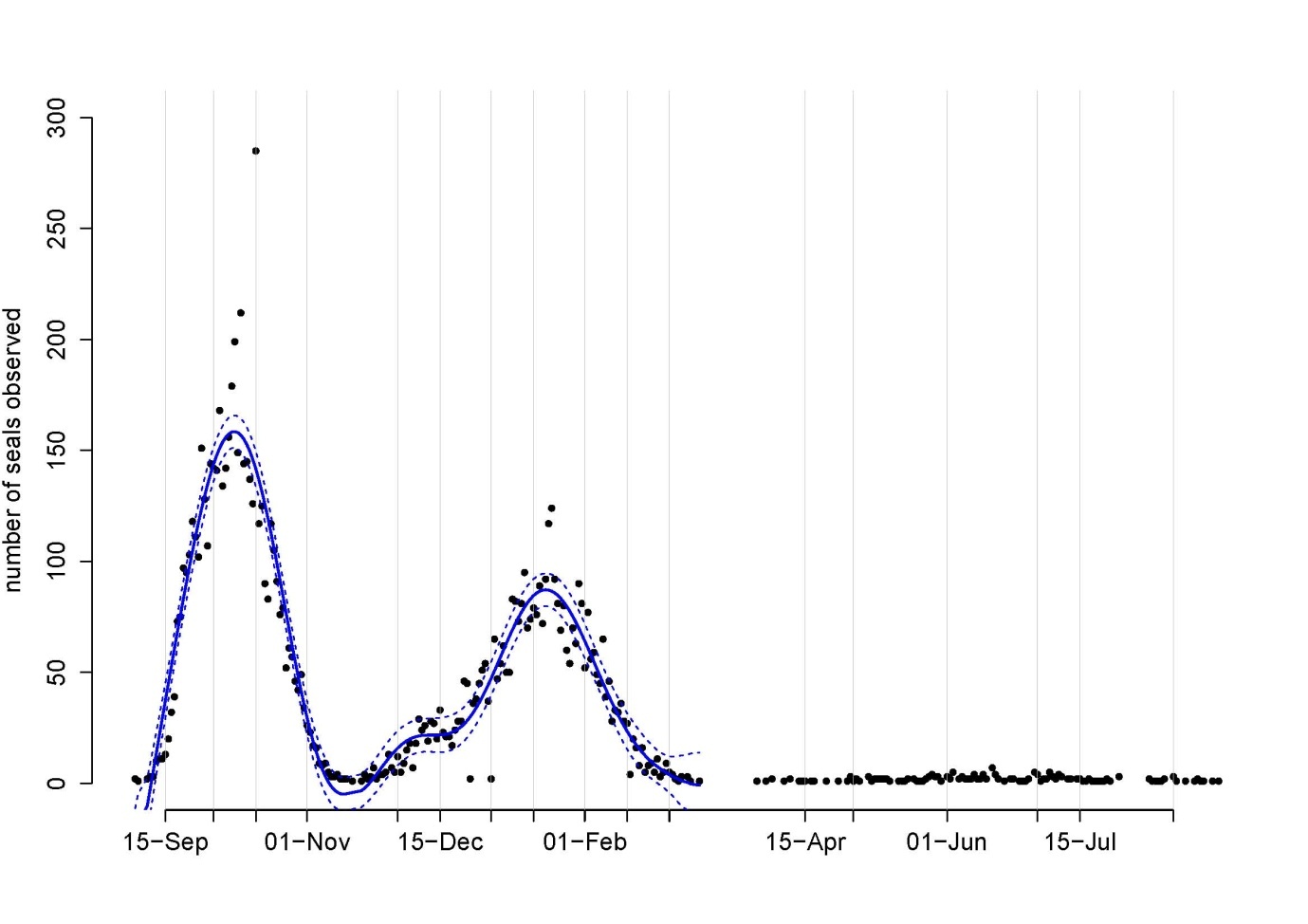
Fig.1.** Number of female elephant seals of 4 years of age resighted ashore at Macquarie Island over a 60 years period (1951-2011). The solid line represents the curve generated by the generalized additive model and the dotted lines represent the upper and lower bounds of the confidence interval.

We used the lower limit of the confidence interval to determine the dates at which the breeding season and the moulting period started and ended. We considered that the date for which this curve passed through the point 0 (i.e. number of seals seen ashore = 0) was the date limiting the breeding season or the moulting period (respectively) (Fig.2, table 1).

**Fig. 2.** Breeding season and moulting period for 4-year old female elephant seals at Macquarie Island.
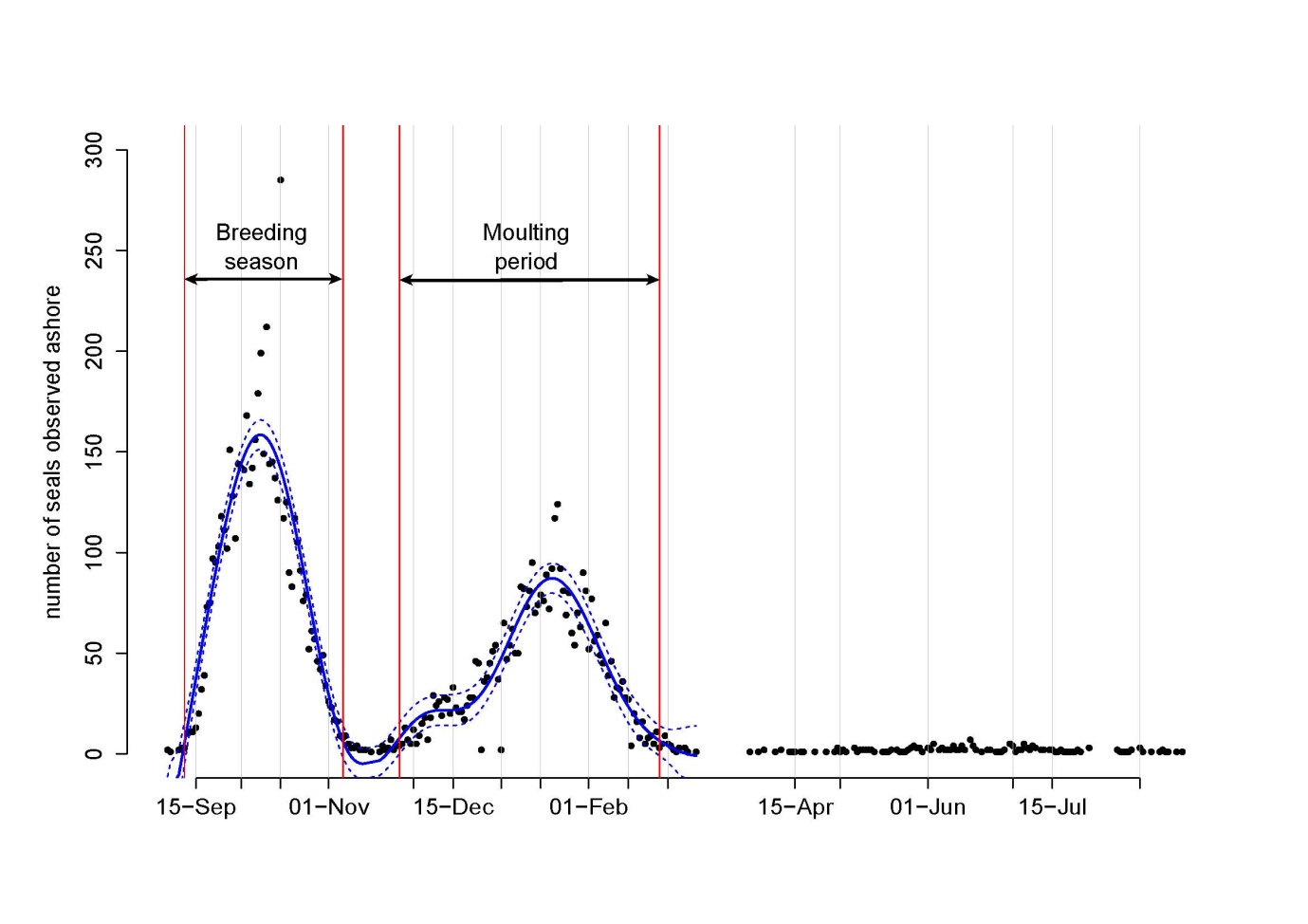


**Table 1.** Dates delimiting the breeding season and the moulting period of adult females elephant seals according to their age. Dates of the breeding season for the 3-year old females could not be determined because of the very low number of 3-year old adults resighted.

| **Age** | **Breeding season** | | **Moulting period** | |
| --- | --- | --- | --- | --- |
|  | **Start** | **End** | **Start** | **End** |
| **3** | - | - | 02 - 11 | 14 - 02 |
| **4** | 11 - 09 | 06 - 11 | 26 - 11 | 26 - 02 |
| **5** | 08 - 09 | 15 - 11 | 07 - 12 | 26 - 02 |
| **6** | 10 - 09 | 18 - 11 | 08 - 12 | 26 - 02 |
| **7** | 10 - 09 | 17 - 11 | 12 - 12 | 27 - 02 |
| **8** | 09 - 09 | 13 - 11 | 20 - 12 | 23 - 02 |
| **9** | 11 - 09 | 07 - 12 | 28 - 12 | 21 - 02 |
| **10** | 11 - 09 | 16 - 11 | 18 - 12 | 23 - 02 |
| **11** | 13 - 09 | 14 - 11 | 21 - 12 | 18 - 02 |
| **12** | 14 - 09 | 16 - 11 | 26 - 12 | 20 - 02 |

Female elephant seals give birth to their pups on average 4 days after their return to land. The minimum period of lactation before the pup weaning is 21.6 days ([McMahon and Hindell 2003](#_ENREF_3)). Females need to spend a minimum of 70 days at sea before the start of their moult ([Hindell et al. 1991](#_ENREF_2)) to rebuild their fat reserves. Consequently, the minimum return date to land for a breeder is the date of the beginning of the breeding season plus 96 days (table 2).

**Table 2.** Minimum return date to land of adult females according to their age. We did not consider individuals older than 7 years because their minimum return date was before the beginning of the moulting season as determined previously.

| **Age** | **Minimum return date to land for moulting** |
| --- | --- |
| **3** | 13 - 12 |
| **4** | 16 - 12 |
| **5** | 13 - 12 |
| **6** | 15 - 12 |
| **7** | 15 - 12 |

For each age class, we considered that all individuals seen ashore during the breeding season were "adults". All individuals resighted on land between the end of the breeding season and the minimum return date to land were considered "juveniles". All individuals seen ashore outside of these periods were considered "unknown". All females considered 'adults' on one occasion were then considered 'adults' for the rest of their life..

For 3-year olds, only individuals seen with a pup were considered adults as we could not determine the dates of the breeding season. To determine the minimum return date to land, we used the minimum start date of the breeding season of all age classes (i.e. 08/09).

Individuals older than 7 years of age were only considered "adults" or "unknown".

***References***

Hindell, M. A. and H. R. Burton. 1988. Seasonal Haul-out Patterns of the Southern Elephant Seal Mirounga-Leonina L. At Macquarie Island Australia. Journal of Mammalogy **69**:81-88.

Hindell, M. A., H. R. Burton, and D. J. Slip. 1991. Foraging Areas of Southern Elephant Seals Mirounga-Leonina as Inferred from Water Temperature Data. Australian Journal of Marine and Freshwater Research **42**:115-128.

McMahon, C. R. and M. Hindell. 2003. Twinning in Southern Elephant Seals: The Implications of Resource Allocation by Mothers. Wildlife Research **30**:35-39.

Wood, S. 2006. Generalized Additive Models: An Introduction with R. Chapman & Hall/CRC.
